# A three component model for superdiffusive motion effectively describes migration of eukaryotic cells moving freely or under a directional stimulus

**DOI:** 10.1371/journal.pone.0272259

**Published:** 2022-08-02

**Authors:** Elvira Toscano, Leandra Sepe, Giusy del Giudice, Rossella Tufano, Giovanni Paolella

**Affiliations:** 1 Dipartimento di Medicina Molecolare e Biotecnologie Mediche, Università degli studi di Napoli “Federico II”, Napoli, Italy; 2 Ceinge Biotecnologie Avanzate, Napoli, Italy; PLOS, UNITED KINGDOM

## Abstract

Although the simple diffusion model can effectively describe the movement of eukaryotic cells on a culture surface observed at relatively low sampling frequency, at higher sampling rates more complex models are often necessary to better fit the experimental data. Currently available models can describe motion paths by involving additional parameters, such as linearity or directional persistence in time. However sometimes difficulties arise as it is not easy to effectively evaluate persistence in presence of a directional bias. Here we present a procedure which helps solve this problem, based on a model which describes displacement as the vectorial sum of three components: diffusion, persistence and directional bias. The described model has been tested by analysing the migratory behaviour of simulated cell populations and used to analyse a collection of experimental datasets, obtained by observing cell cultures in time lapse microscopy. Overall, the method produces a good description of migration behaviour as it appears to capture the expected increase in the directional bias in presence of wound without a large concomitant increase in the persistence module, allowing it to remain as a physically meaningful quantity in the presence of a directional stimulus.

## Introduction

Eukaryotic cells growing on culture plates in standard conditions move in all possible directions travelling smaller or larger distances, depending on cell type. Cell movement is usually described by using numerical parameters and mathematical models provide powerful options to better understand cell behaviour and to test hypotheses. The diffusive model is possibly the simplest model used to describe cell movement and is based on the assumption that cells move freely without a preferential direction and the probability of changing direction is the same at sampling each time. The diffusive model also implies that each movement is independent from that of the previous time steps, and that mean squared displacement (MSD) is proportional to elapsed time, according to a diffusion coefficient (D). This model is mostly accurate when suitably long observation times are used [1, 2]. The diffusive model may be extended to include additional motion patterns by introducing an *α* exponent (*MSD* = *kt*^*α*^) which, for a purely diffusive movement, is equal to 1. The *k* parameter assumes different meanings for different *α* values, specifically for *α = 1 k = 2dD*, where *d* is the number of dimensions, while for *α = 2 k = v*^*2*^ as in uniform linear motion, where *v* represents the speed. Values of *α* ranging between 1 and 2, define various types of superdiffusive movements, such as those produced by the presence of directional bias and/or persistence [3, 4]. Directionally biased movement is typically observed in presence of a molecular gradient, such as that generated by a nutrient source, an attractant factor, or even a wound inflicted to the cell layer, and may be modelled as a factor able to alter diffusive movement such as that produced by a random walk [5] by using the equation:

$$MSD=u^{2}t^{2}+2dDt$$ (1)

where *u* is the speed increase over the purely random movement. Persistent migration, such as that similarly produced by limiting the direction change in a random walk [5], had been already observed in cultured cells in the seventies, when mouse fibroblasts have been found to persist in their direction for about 2–3 hours [2]. Years later, Selmeczi et al., by evaluating the parameters obtained from the model for both human fibroblasts and keratinocytes, proved that these cells maintain memory of the past movement [6]. Persistence is especially observed when movement is sampled at relatively short time periods, and appears as a sort of resistance to a change of direction, possibly deriving from the need for membrane/cytoskeletal reorganisation. Both directional bias and persistence produce a superdiffusive movement with a tendency to persist in the direction of motion. However, in the case of persistence, the followed path remains globally unbiased, meaning that there is no overall preferred direction if movement is not otherwise biased. This feature, which generates a tendency to maintain, at each time step, the direction of the previous one, has been variably referred to as persistence, linearity or sometimes also directionality, and differently measured in time units, i.e. how long the current direction influences the movement in subsequent time periods, or in terms of ratio between net displacement and length of the followed path [7, 8]. In the model initially proposed by Fürth et al. in 1920 [9]and described by Alt et al. in 1990 [10], the relation between MDS and time (*t*) is given by the following equation:

$$MSD=2S^{2}P\left[t-P(1-e^{\frac{-t}{P}})\right]$$ (2)

where *S* is the root mean squared speed and *P* is the directional persistence time, i.e. the time in which cell movement tends to persist in the same direction. For t << P, the displacement is determined by purely unidirectional motion and MSD ~ S^2^t^2^; whereas for t >> P, the movement is described by a normal diffusion and MSD ~ 2S^2^Pt [11]. This model is particularly effective in interpreting a wide range of superdiffusive motion patterns, starting from purely diffusive movement, with MSD proportional to time, up to movement along an almost straight line with MSD proportional to squared time as for uniform linear motion.

Several computational tools and methods have been developed over time, aimed to evaluate cell movement features including speed, persistence and directionality. Almost all tools quantify the length of displacements and calculate average speed; in some cases, cell persistence and directionality are also evaluated. However, there are differences in the way the different tools express, also in terms of units, and calculate these parameters: cAveMap [7] and iTrack4U [8] derive persistence from relation of end-to-end distance of a cell trajectory and its total length, while MotoCell [12, 13] and CellMissy single-cell module [14], calculate the same quantity but refer to it as linearity and end-point directionality ratio respectively. Pathfinder [15] describes persistence in terms of the absolute angle of deflection, while in Cell_motility [16] and MotoCell persistence is expressed in time units and is calculated by fitting the persistent model, reported in Formula (2), to MSDs.

This apparent confusion reflects the tight connection between directional persistence and directional bias: as both increase path linearity and each may influence the evaluation of the other one, wrong quantifications and misinterpretation of cell behaviour may easily occur when, for example, cells move under a strong directional stimulus and both movement features are present at the same time.

The model proposed in this study improves on that, by describing cell displacement as the vectorial sum of three vectors, corresponding to random, persistence and bias components. It was successfully tested on a large set of data from simulated and experimental cells. The procedure developed on the basis of that model can effectively analyze complex cell movement and was shown to be effective in resolving the various motion features in different combinations.

## Materials and methods

### Cell culture

Cells were grown in 100 mm diameter Petri plates in Dulbecco’s Modified Eagle’s Medium (DMEM) supplemented with 10% fetal bovine serum (FBS), penicillin (10 U/ml), streptomycin (10 ng/ml) and L-Glutamine (2mM) and maintained in incubator at 37°C and with atmosphere made up to 95% air and 5% CO2. Cell propagation was performed by detaching cells with a solution of trypsin/EDTA (trypsin 0.05% and 0.53 mM EDTA) and collecting them with complete culture medium. After centrifugation at 1200 rpm for 5 minutes, pellets were suspended in fresh medium, properly diluted, and plated again.

The cell lines used for time lapse acquisitions include murine fibroblasts NIH-3T3 [17] and NIH-Ras produced by transfecting RasV12 into NIH-3T3 as also reported in previous articles [18], and human immortalized cell lines HeLa from cervical cancer [19], T24 from bladder carcinoma [20] and MDA-MB-231 from breast cancer [21].

### Motility assay

To investigate the random movement ability, 25000 cells/well were seeded in 12 well plates and maintained in complete medium at 37°C in an incubator with 5% CO_2_. After 16–18 hours, the plate was placed in the incubator chamber of the microscope. For wound healing assays, cells were seeded in confluent monolayers by plating 250000 cell/well in 12 well plates in complete medium; 24 hours after plating the cell layer was scratched with sterile pipette tip.

### Data acquisition

Phase contrast images (objective 10x) of different samples have been acquired every 10 minutes for 24 hours by using the Zeiss Cell Observer system composed by an inverted microscope (Axiovert 200M), an incubator chamber that maintains the temperature at 37°C and CO2 pressure at 5%, and a digital camera (Axiocam H/R). A motorised stage along the three axes permits prolonged automatic acquisitions at different positions. For this work, digital frames were acquired as 8 bit images of 650x514 pixels. The pixel scale of the acquired images is 0.767 pixel/μm, obtained by acquiring with the same system an image of a Burker chamber with known measures.

Cell displacements have been tracked by using a semi-automated procedure available within MotoCell [12]. The tracking procedure allows to collect cell positions (in terms of x/y coordinates) at different times (frames) to construct the entire path of each cell that is characterised by an origin (start, newborn, found, gone in) and a destiny (split, dead, lost, gone out). The registered data are written and stored in a text file that can be read by MotoCell to perform the quantitative analysis.

### Data analysis

Mathematical, statistical and graphical analyses have been carried out within the R environment [22], using the core functions as well as the following packages: *graphics* and *stats* from the basic configuration and *ggplot2* [23]. Functions provided within the R environment were used either directly through the RStudio development environment [24] or by calling them within MotoCell analysers through the Rserve [25]. Curve fitting by R function *nls* (non-linear least squared) was done either by using the provided models or others described by a custom equation, as reported in text.

Diffusive behaviour was quantified, within MotoCell, on the basis of mean squared displacement (MSD) and time, by using the “Diffusion” module which fits the function *MSD* = *kt*^*α*^ to data, assigning to each value a weight proportional to the number of averaged squared displacements. Persistence analysis was carried out by using the “Persistence” module, based on Formula (2). The mean squared displacements were calculated by collecting and squaring for each path the displacements corresponding to all time intervals between 40 minutes and the full path duration, and then by averaging the squared displacements for each interval.

Time course analyses were performed by separately analysing overlapping time windows of different length, each spanning a fraction of the total duration of the experiment. Most analyses were automated by writing PHP scripts and executing them within the MotoCell environment.

### Generation of simulated cell populations

Simulated datasets used to test the developed procedure were generated by using an *in silico* simulation system developed for internal uses and not yet published. Briefly, the tool is accessed through a web application used to provide input data, including cell parameters and general features of the experiment, through a dialog box; output data are collected in a text file which records an “experiment” as the results of the simulation of each cell of a given “plate” at each time point. The simulation system mimics the behaviour of a cell population by individually simulating each cell as a stochastic entity acting according to defined models representing the main cellular processes, such as growth, proliferation, migration and death. The datasets used in the present work were produced by only using and taking into account movement-related features. For each simulated moving population, a bias vector and/or a persistence module are defined, thus producing cell migration patterns ranging between completely diffusive, persistent or biased, in various combinations. A cell displacement in a given time interval (typically 40 minutes) is simulated as the resultant of a random vector, a persistence vector with a user defined module and the same direction as the previous cell displacement, and a user defined bias vector. No directionally biased and zero persistence movement was obtained by zeroing the corresponding vector. The random component was obtained from a brief random walk of n random direction sub-steps with duration corresponding to 1/nth of the time interval and MSD equal to 1/nth of the requested MSD; for the datasets used in the present work, the chosen value (n = 10) was selected as a good compromise between computation time and even distribution of step lengths thus avoiding synthetic paths to be composed by random vectors identical in module and differing only for direction. The simulation procedure with the steps involved has been provided as pseudocode in S2 File.

## Results

### 1. Persistence and bias conflict in cell movement analysis

The movement of five different cell lines was followed while growing under standard culture conditions and in wound healing experiments, i.e. while recovering from a wound inflicted to the cell layer, a condition usually associated with directional motion, stimulated by the inflicted wound. The selected cell lines represent a spectrum of mammalian cell types with a range of growth features and migration patterns and include HeLa, MDA-MB-231 and T24, which are human transformed cell lines isolated from tumours with high metastatic power, NIH-3T3 an established untransformed cell line from murine embryonal fibroblasts and NIH-Ras, a cell line strongly tumorigenic in nude mice with high dissemination potential, obtained by overexpressing in NIH-3T3 the same oncogene (a constitutively active form of Ras) known to be present in T24 cells. Images of each cell culture were acquired by time-lapse microscopy and cell movement was characterised in terms of average displacement length per 40 minute interval, diffusion parameters *α* and *k*, persistence parameters *s* and *p* and directional bias vector module and angle (Table 1) using the procedures described under methods. Average distance was determined by simply averaging the lengths of all cell displacements observed during each 40 minute interval and varies between 5 and 18 μm according to cell type and experimental conditions. When tested for diffusive motion, all cell lines exhibited a superdiffusive behaviour, with an *α* coefficient well above 1, which becomes higher, often close to 2 in presence of a wound. The observed superdiffusive movement is typical of most adherent cell lines and it may usually be explained by a combination of directional persistence, related to focal contacts between cell and culture surface [2, 26, 27], and a directional bias (raw bias), typical of wound healing experiments. Persistence analysis in Table 1 shows that a degree of persistence (expressed in time units) is always present, with the highest values observed during wound healing. The raw bias, calculated by averaging all cell displacement vectors, has a very low module (typically up to around 10% of the average distance) in randomly moving populations and becomes much larger after a wound. In wounded populations, all cells were observed to move towards the empty space, as indicated in column *δ*, where the difference is reported between the direction of the raw bias vector and the angle expected from the position of analyzed cells in relation to the wound (0 or 360 degrees for cells located on the left side of a vertically oriented wound, 180 degrees for cells on the right side of it); for randomly moving populations, delta (*δ*) was not calculated because no expected angle may be defined for these samples. Analysis of bias and persistence parameters in wound healing experiments shows that cell populations with high raw bias also had high persistence, suggesting a possible interference. To confirm this interpretation with more controlled data, three datasets were produced from simulated cell populations corresponding to different experimental situations, where persistence and bias contributions to overall migration were set a priori. For each simulated population, cell paths were generated according to a purely diffusive pattern, modified by adding a fixed amount of persistence and/or directional bias (see materials and methods). Under these conditions, the input values used to simulate movement, were assumed to be the expected values when examining the output results. To generate movement patterns in the same range as the experimental ones, the simulation was carried out using an MSD of 100 μm^2^, expected to produce an average displacement similar to that observed for NIH-3T3 fibroblasts, while the directional bias was set to 8 μm. Regarding persistence, an 8 μm value was chosen by trial and error, as it was shown to produce, in directionally unbiased populations, a level of persistence corresponding to about 65 minutes, i.e. well within the range of values observed for the experimental populations. The results are reported in the last three rows of Table 1: cells simulated with no directional bias (*p* = 8 μm and *b* = 0 μm, marked as *persistent* in the Table 1), showed, as expected, a persistence time (*p*) of 64 minutes and an almost null raw bias vector; for cells following zero persistence motion (*p* = 0 μm and *b* = 8 μm, marked as *biased)*), a raw bias vector was detectable as expected, but the obtained persistence time was much higher than the expected null value. For more complex movements, when both bias and persistence were present at the same time (line *pers*.*+ biased*, *p* = 8 μm and *b* = 8 μm), raw bias module and persistence time were both substantially higher than the expected 8 μm (bias) and 65 minutes (persistence).

**Table 1:** Superdiffusion of different cell lines in different culture conditions.

cell population		average distance (μm) per 40’	diffusion		persistence		raw bias	
condition	line		k (μm^2^/min^α^)	α	s (μm/min)	p (min)	module (μm)	δ (degrees)
random	NIH-3T3	11.3	5.8 ± 2.0	1.14 ± 0.06	0.42 ± 0.06	46 ± 14	0.4	-
	NIH-Ras	14.8	4.6 ± 1.7	1.25 ± 0.04	0.47 ± 0.07	58 ± 20	1.6	
	T24	17.9	4.2 ± 0.7	1.32 ± 0.03	0.52 ± 0.05	65 ± 14	0.8	
	HeLa	6.8	0.4 ± 0.07	1.37 ± 0.03	0.23 ± 0.05	40 ± 19	0.4	
	MDA-MB-231	5.3	0.2 ± 0.1	1.56 ± 0.09	0.22 ± 0.04	75 ± 36	0.4	
wound	NIH-3T3	9.5	0.1 ± 0.0	1.81 ± 0.05	0.28 ± 0.02	307 ± 85	6.0	13
	NIH-Ras	14.9	0.5 ± 0.1	1.76 ± 0.04	0.40 ± 0.01	479 ± 64	10.8	19
	T24	9.7	0.4 ± 0.1	1.61 ± 0.04	0.33 ± 0.03	134 ± 33	6.0	11
	HeLa	6.6	0.02 ± 0.01	2.00 ± 0.10	0.23 ± 0.05	133 ± 90	4.0	6
	MDA-MB-231	6.4	0.7 ± 0.1	1.33 ± 0.03	0.24 ± 0.03	55 ± 16	1.2	43
persistent	simulated NIH-3T3	11.8	4.8 ± 0.7	1.18 ± 0.02	0.37 ± 0.01	64 ± 3	1.2	-
biased		11.3	0.1 ± 0.0	1.88 ± 0.01	0.33 ± 0.05	143 ± 55	7.7	1
pers.+ biased		16.8	0.2 ± 0.0	1.94 ± 0.01	0.46 ± 0.04	641 ± 356	14.0	0

It appears that by following this approach, bias and persistence cannot always be clearly distinguished and, in the case of combined bias and persistence, both tend to be overestimated.

### 2. A combined model to study movement of cultured cells

**Fig 1:**
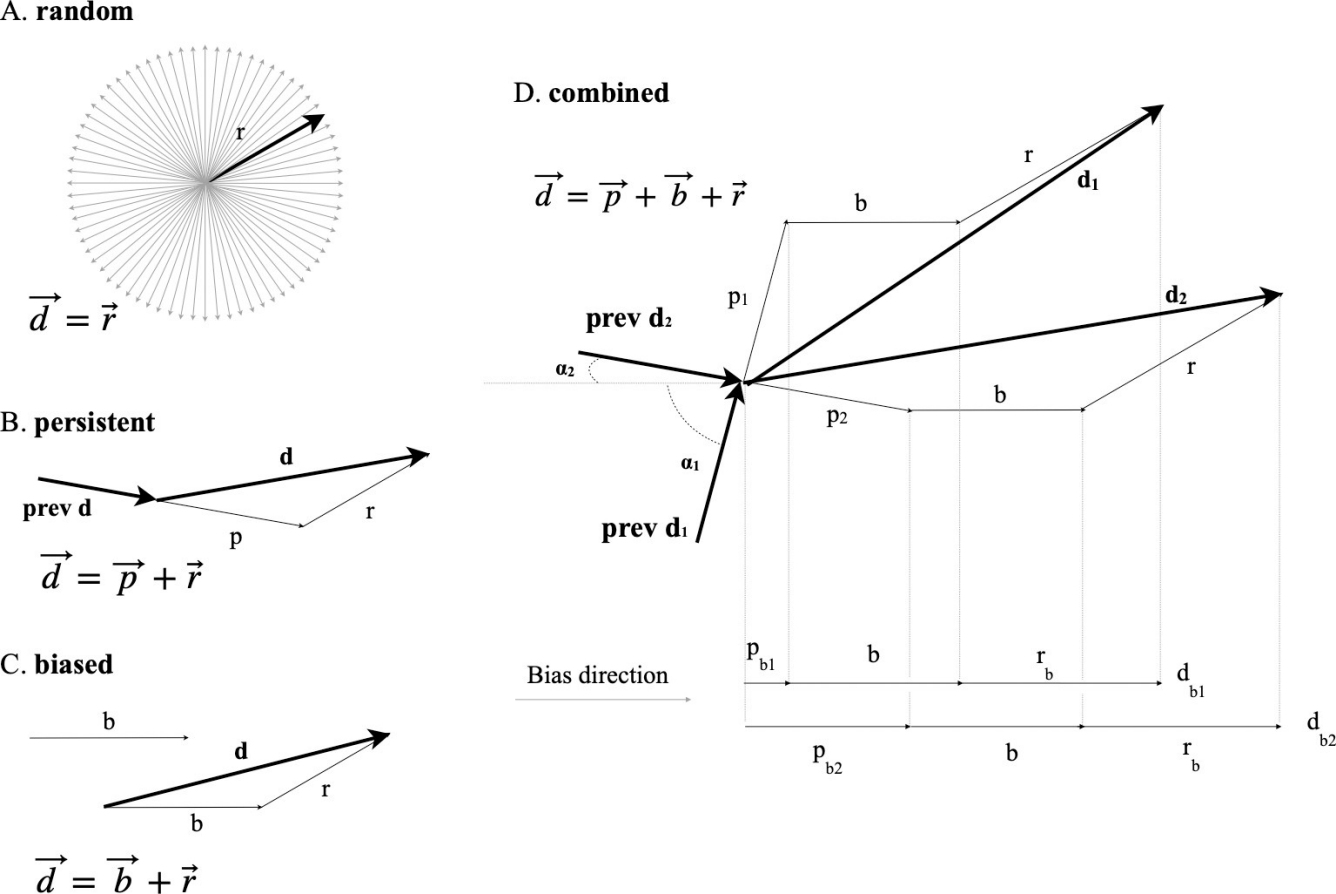
A three component model for single cell movement. In the case of purely diffusive motion (A), cell displacement is modelled as a random vector of radius r. Persistent and biased movement (B and C), respectively add a persistence (*p*) or bias (*b*) vector to the random one. In the general case of combined movement (D), displacements are a combination of the three vectors and the directional component (d_b_) consists of the whole bias module with the addition of random and persistence contributions (respectively r_b_ and p_b_). The persistence contribution depends on the difference between bias and persistence angle (*α* angle); its length increases, according to the cosine function, when the *α* angles decreases.

To address the previously described issue, cell motion was modelled as a combination of three vectors: random (*r*), persistence (*p*) and bias (*b*), which can vary according to cell line and culture conditions, ranging from simple to more complex combinations, where all the three vectors contribute to the overall migration (Fig 1). In a purely random movement (Fig 1A), each displacement *d* consists of a random vector which can take all possible orientations; in the case of persistent movement (Fig 1B), the final displacement *d* derives from a persistence vector having the same direction as the previous movement (*prev d*), added to the previously described random vector; a biased displacement is modelled as the sum of a random and a bias vector, which, for all cells, is assumed to be oriented along the same direction, for example towards an attractant or according to a directional stimulus (Fig 1C). Under experimental conditions, all three vectors are usually present (Fig 1D): in this case, displacement (*d*) is the vectorial sum of the bias (*b*), random (*r*) and persistence (*p*) vectors. Given two different displacements, d_1_ and d_2_, with the corresponding previous ones (*prev d*_*1*_ and *prev d*_*2*_), and a bias direction, for each of them, the displacement along the bias direction (*d*_*b*_) is the sum of the whole bias module (*b*) and the random and persistence contributions (*r*_*b*_ and *p*_*b*_), i.e., the projections of random and persistence vectors onto the bias direction. Random contributions (*r*_*b*_) to d_b_ differ among the steps of a path as well as among cells and should be considered as "noise" which tends to zero for a large number of displacements. Persistence contribution (*p*_*b*_) depends instead on the difference between bias and persistence angle (*α* angle) according to the formula:

$$p_{b}=p\cdot cos(\alpha )$$ (3)

which means that the smaller the *α* angle, the higher is the contribution of persistence to directionality. We graphically explain this relation in Fig 1D where displacements d_1_ and d_2,_ projected on the bias direction, differ for *p*_*b1*_ and p_b2_ lengths that depend in turn on *α*_*1*_ *and α*_*2*_ angles. Thus, the directional component of cell movement as a function of persistence and bias may be defined according to the following function:

$$d_{b}=b+p\cdot cos(\alpha )$$ (4)

**Fig 2:**
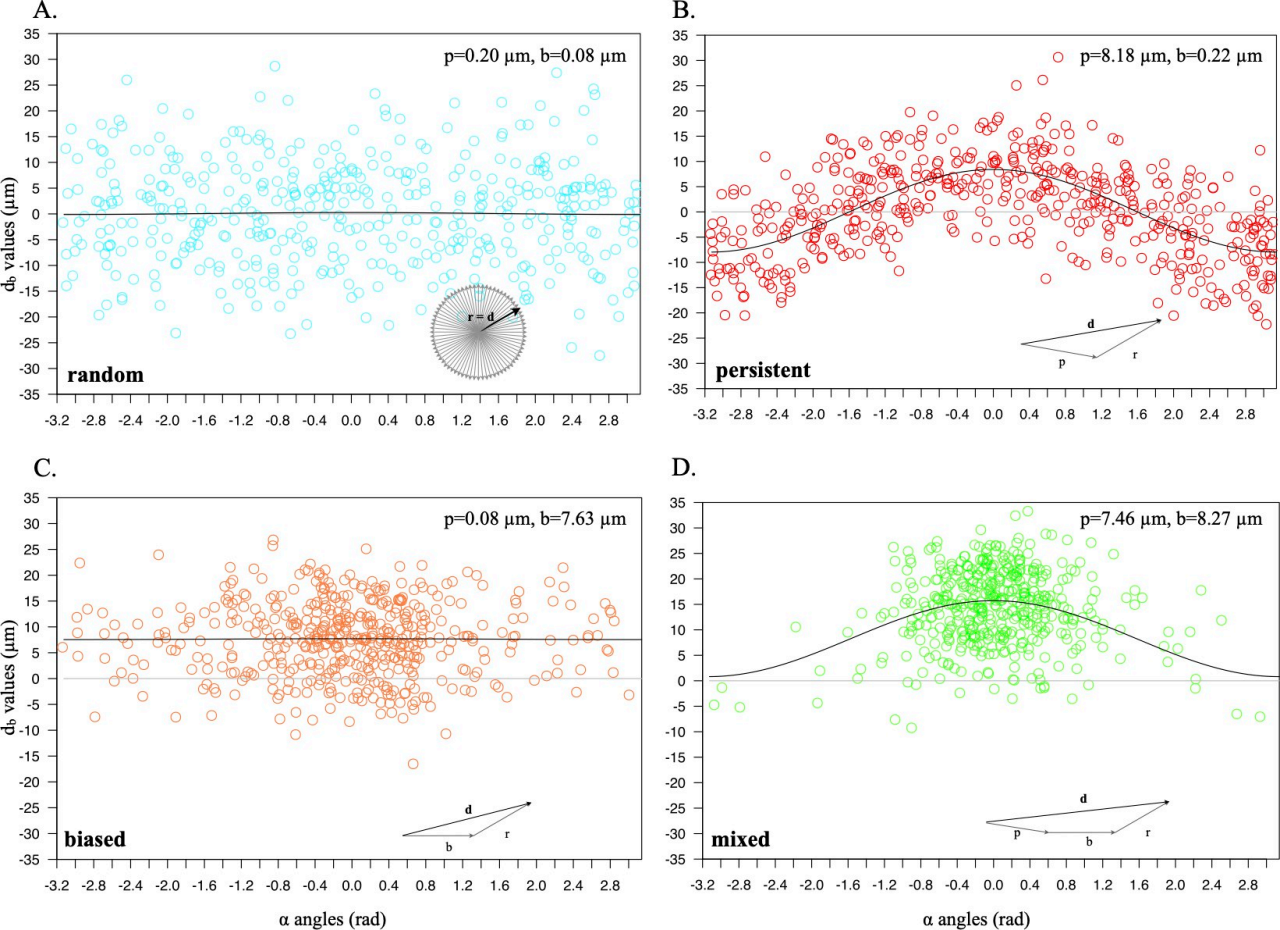
Persistence and bias effect on directional component of cell movement. The directional components of cell displacements from cell populations are plotted (y axis) against their *α* angles (x axis); (A, B) correspond to populations generated by simulating random (*p* = 0 μm; *b* = 0 μm), persistent (*p* = 8 μm; *b* = 0 μm), biased (*p* = 0 μm; *b* = 8 μm) and mixed movements (*p* = 8 μm; *b* = 8 μm). The continuous line corresponds to curves identified by fitting the described model to the data; the numerical parameters are shown on the top of each graph.

According to the model, longer d_b_ values are obtained for *α* angles closer to zero, because they include a higher persistence portion. To test whether this relation may be detected in simulated NIH-3T3 cells, different pairs of *p* and *b* parameters were used to produce simulated populations and analysed by plotting, for all displacements, directional component lengths against *α* angles. Results in Fig 2 show that for random movement, obtained with *p* = 0 μm and *b* = 0 μm (Fig 2A), *α* angles are homogeneously distributed between -π and +π, while *d*_*b*_ values are symmetrically distributed around zero for all angle values. This is also observed when cell movement is simulated with no bias component, i.e. *p* = 8 μm and *b* = 0 μm (Fig 2B), but in this case d_b_ values appear to follow a cosine curve, with maximal values for α = 0 and minimal ones for α = ± π. When bias is present, with *p* = 0 μm and *b* = 8 μm (Fig 2C), displacement angles are concentrated around the bias direction (α = 0) but, for all angles, *d*_*b*_ values are on average offset by a factor corresponding to the bias module. When both bias and persistence are added to the motion, *p* = 8 μm and *b* = 8 μm (Fig 2D), the effects are independently visible as d_b_ values follow the cosine function and are at the same time offset according to the bias. The previously described Formula (4) was used to fit the data in all cases; the curves, reported as continuous lines in each graph, were produced by using the calculated persistence and bias parameters (shown on the top/right for each panel).

**Fig 3:**
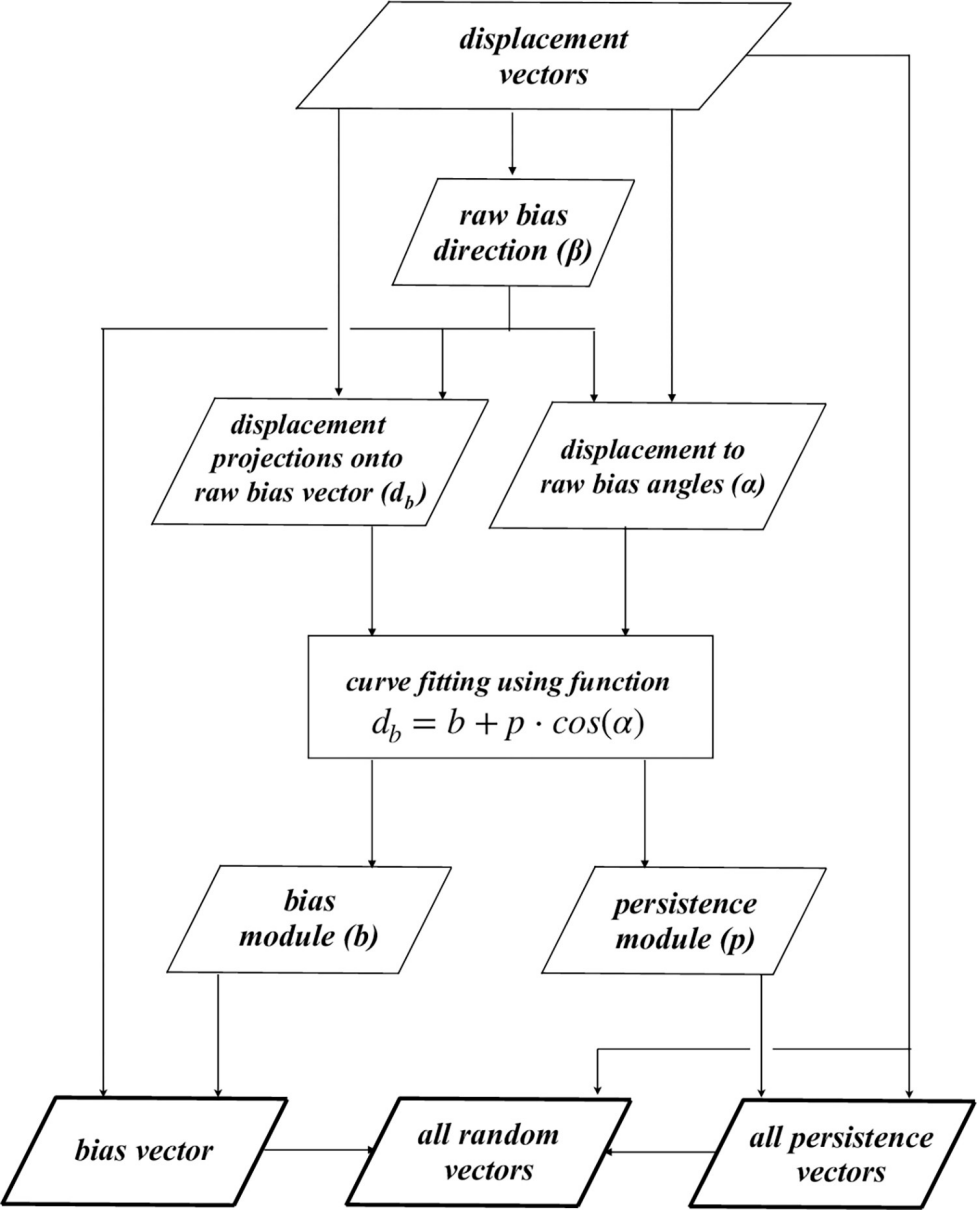
Schematic representation of the procedure for parameters evaluation. This figure schematically reports the procedure that, starting from displacement vectors, leads to the calculation of the bias vector, the persistence module and the list of the random vectors deprived of bias and persistence components.

On the basis of these results, the proposed model was considered a good candidate to generically describe any migratory behaviour and the procedure schematically synthesized in Fig 3, was set up. First, the raw bias vector calculated as the vector sum of all displacement vectors observed in a given time interval is used to obtain the raw bias direction *β*. After that, for each displacement, the projection onto the raw bias direction (d_b_) is determined, as well as the displacement to raw bias angle (*α*), i.e. the difference between previous displacement and bias direction. The previously defined model (formula 4) is used to fit the directional component lengths (d_b_) as function of *α*, to obtain bias (b) and persistence (p) modules. Finally, having defined bias and persistence modules, bias and persistence vectors are subtracted from each displacement, to obtain the random vectors.

### 3. Validation of the model

The described procedure was validated by analyzing cell displacements from datasets obtained by simulating cell populations characterized by different pre-defined values of bias, persistence and random module. The datasets include the paths followed by 30 cells generated by using bias and persistence values ranging between 0 and 16 μm as indicated in the header row and column of Table 2. Bias (*b*) and persistence (*p*) values evaluated by the described procedure show that in the case of movement with zero persistence (first row) or no directional bias (first column) calculated bias and persistence modules are very close to the expected values.

**Table 2:** Bias and persistence estimated from simulated cell populations.

Persistence (μm)	bias (μm)							
	0		8		12		16	
	*b*	*p*	*b*	*p*	*b*	*p*	*b*	*p*
**0**	0.4 ± 0.3	-0.4 ± 0.5	7.8 ± 0.5	-0.2 ± 0.6	12.3 ± 0.9	-0.2 ± 1.0	16.6 ± 1.2	-1.1 ± 1.4
**8**	0.3 ± 0.3	9.2 ± 0.5	7.8 ± 0.8	7.9 ± 0.9	12.0 ± 2.1	8.3 ± 2.3	13.3 ± 3.8	10.5 ± 4.1
**12**	0.0 ± 0.3	12.1 ± 0.5	6.5 ± 1.4	13.1 ± 1.6	15.3 ± 4.0	9.4 ± 4.0	16.7 ± 4.4	11.3 ± 4.6
**16**	0.2 ± 0.3	16.2 ± 0.4	8.2 ± 2.2	15.7 ± 2.4	11.8 ± 4.3	16.4 ± 4.5	15.4 ± 7.9	16.3 ± 8.1

A similarly good correspondence between expected and measured values was also observed when persistence and bias vectors were both present in combination. The random vectors, calculated for each population, also produced an average module close to the 9 μm/40 min value used as input to the simulation (S1 Table). The same combinations of persistence and bias vectors were also tested for a second simulated set of populations characterized by a larger value of random module (12.5 μm/40 min) and also in this case the obtained values show good correspondence with the expected ones, thus proving that the efficacy of the analysis method is not impaired by changes in the random module (S2 Table). Calculated random vectors produced for each population an average module close to the one used as input (S3 Table).

In order to evaluate the performance of the model in relation to cell population size, the previous analysis was repeated using datasets of size ranging between 10 and 100 cells. The results (S2 Fig) show that for both bias and persistence values, calculated/expected ratios are within ± 0.4 for datasets of 10 to 20 cells but quickly go down to smaller ones for 30–50 cells and are reduced to within ± 0.1 for bigger datasets (100 cells). In addition, median values, which are very variable for 10 and 20 cells, starting from 30 cells, i.e. the population sizes used in our evaluations, become less variable and closer to the expected values.

The model was also used to evaluate the datasets reported in Table 1, to assess its effectiveness in separating bias and persistence components also with experimental populations. The results are reported in Table 3 as random, persistence and bias vector modules for a 40 minute time interval; for wound healing experiments, angle *δ*, i.e. the angle between bias vector and expected migration direction, is also reported. For all cell lines, in absence of wound stimulus, the detected bias has values close to zero and movement, as might be expected, is essentially determined by random and persistence module. Cells with larger average distances show correspondingly higher values for both the random and the persistence module. For all cell lines, the introduction of a wound stimulus results in a modified movement pattern, characterized by a bias vector of considerably higher module than that observed in absence of wound for the same cell lines and oriented along the expected direction, i.e. towards the empty space left by the wound, as indicated by *δ* angle values ranging between 2 and 43 degrees.

**Table 3:** Characterization of movement of different cell lines in different experimental conditions.

cell population		average distance (μm) per 40’	random module (μm)	persistence module (μm)	bias	
condition	line				module (μm)	δ (degrees)
random	NIH-3T3	11.3	10.4	4.9 ± 0.6	0.3 ± 0.4	-
	NIH-Ras	14.8	13.2	8.2 ± 0.9	1.1 ± 0.7	
	T24	17.9	15.6	10.7 ± 1.2	1.1 ± 0.9	
	HeLa	6.8	6.7	1.6 ± 0.4	0.5 ± 0.2	
	MDA-MB-231	5.3	5.5	2.0 ± 0.5	0.0 ± 0.3	
wound	NIH-3T3	9.5	7.8	1.5 ± 0.7	5.7 ± 0.6	22.47
	NIH-Ras	14.9	11.1	7.0 ± 2.1	6.1 ± 1.8	19.21
	T24	9.7	7.4	3.3 ± 0.7	4.3 ± 0.6	2.40
	HeLa	6.6	5.9	1.5 ± 0.7	2.9 ± 0.6	5.68
	MDA-MB-231	6.4	6.8	2.8 ± 0.4	1.4 ± 0.3	42.73

**Fig 4:**
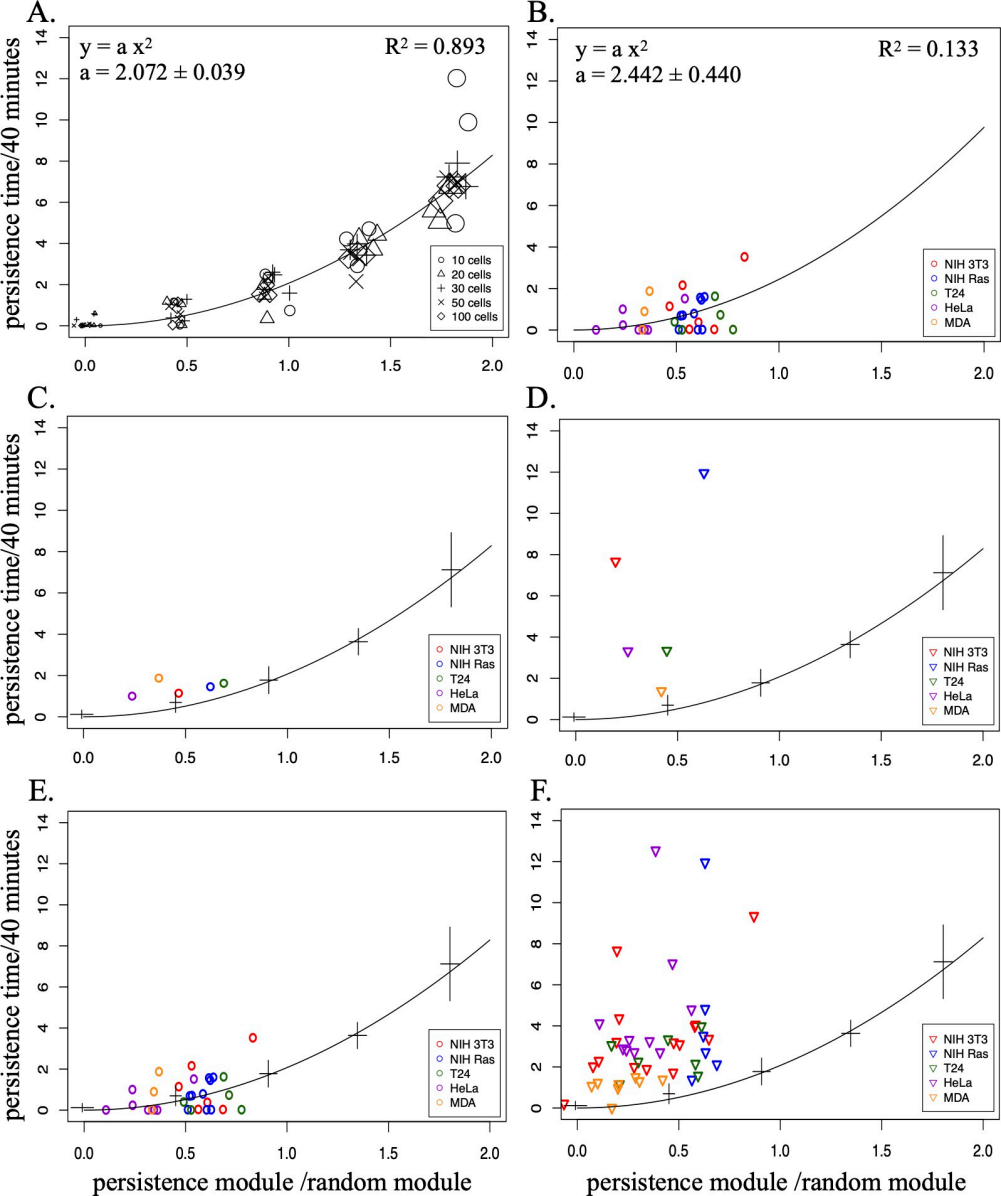
Relation between persistence evaluated as time or as vector length. Persistence values calculated by using Formula (2) or the proposed model: results are compared by plotting, for each dataset, the resulting persistence times, normalized against the time interval (40 minutes), versus the persistence module, normalized against the corresponding random module. (A) Persistence values calculated for datasets containing 10 (circle), 20 (triangle), 30 (plus), 50 (cross) or 100 (diamond) cells simulated at different persistence levels (0, 4, 8, 12 and 16 μm) and reported in the plot as symbols of increasing sizes. For each persistence level, three replicated datasets were produced for each cell number. Fitting the indicated quadratic function to the data produced the “a” parameter value and the R^2^ determination coefficient reported at the top. The black line represents the curve defined by the calculated “a” parameter. (B) Persistence values calculated as in (A) from NIH-3T3 (red), NIH-Ras (blue), T24 (green), HeLa (violet) and MDA-MB-231 (orange) cells moving in absence of a wound stimulus. The black line corresponds to the curve calculated by fitting the quadratic function to the experimental data as in (A). (C, D) Persistence values of unwounded (C) and wounded (D) experimental populations of Tables 1 and 3, plotted after the described normalization step and using as a reference the curve calculated in (A); the crossed bars indicate means and standard deviations of the values used to produce the curve. (E, F) The same as in (C, D) for a larger number of experimental populations.

It should be noted that the present model, unlike others which typically express persistence as a time, uses a persistence vector which, combined with a random one, produces the final displacement. This difference in expressing persistence makes it not immediate to compare results, as both values are in some way affected by the used time interval or the distance covered during it. To better understand the relationship between the two methods, persistence expressed as time and as vector module were plotted compared with each other, after normalizing both by dividing the first by the time interval (40 minutes in this case) and the second by the module of the calculated random vector. When persistence values from simulated datasets are plotted in this way, the relation appears to follow a quadratic trend (Fig 4A) which remains the same with datasets of different numerosity and different levels of persistence (0, 4, 8, 12, 16 μm). By using a simple quadratic equation for curve fitting, the resulting curve closely follows the data points with a determination coefficient R^2^ very close to 1. Similar results are obtained (Fig 4B), when “time” vs “space” persistence values are calculated for experimental datasets, obtained from cells moving in absence of known directional stimuli: also in this case the relation appears to follow a quadratic curve very close to the one determined from the simulated data, and characterized by a second power coefficient very close to 2.0 as before, although with a lower R^2^ value.

Using as a reference the curve calculated in Fig 4A, the persistence values calculated for experimental populations and reported in Tables 1 and 3 were plotted against each other after the normalisation step described above, to comparatively evaluate the two methods. All the data points from unstimulated populations (Fig 4C) remain close to the reference curve, much more than data points obtained from wounded cells (Fig 4D), which are consistently away from and above the reference curve, thus confirming the previous assumption that the presence of a directional bias is significantly altering the calculated values of time persistence. Similar results are obtained when the same analysis is carried out on a much larger number of experimental populations, as shown in Fig 4E and 4F.

### 4. Experimental dataset analysis

**Fig 5:**
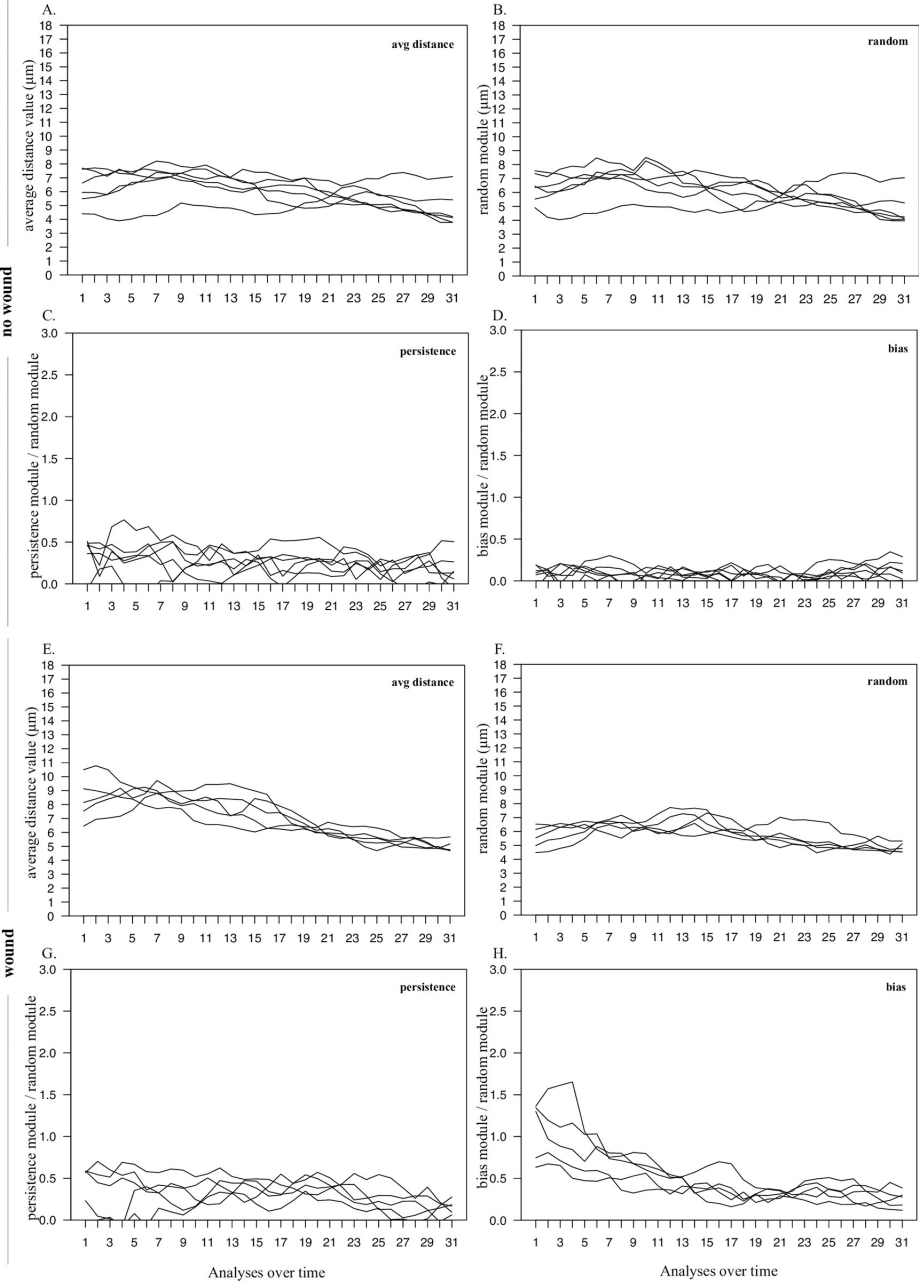
Movement components of HeLa populations over time. HeLa cell movement on a culture plate was evaluated both in absence (A-D) and in presence (E-H) of a wound stimulus. The line plots correspond to independent cell populations; for each of them, the plots report average displacements modules measured over 40 minute steps (A and E), as well as random (B and F) persistence (C and G), and bias (D and H) values, calculated from the observed displacements. Persistence and bias modules were normalized to the corresponding random module. All the values were evaluated at 40 minute intervals using the data from overlapping four hour windows.

The described model was used to study movement trends in time and the evolution of its components in HeLa cell populations moving in standard cultures as well as after a wound stimulus. In absence of directional stimulus (Fig 5A–5D), all values were essentially stable over time, with movement mainly characterized by random and persistence, while bias remains very low during the whole time. In presence of a wound, higher average distance values were observed (Fig 5E), especially in the time windows immediately following the wound stimulus. At later times, the observed distances tend to be reduced, probably because of the concomitant progressive closure of the wound space, clearly visible in the images acquired at different time points during the experiment (an example is reported in S1 Fig). The wounded populations showed strong bias, with the highest values at the beginning of the observation time; as time goes by, the bias module tends to be reduced following a trend similar to that observed for the average distance (Fig 5H); the random module does not appear to change accordingly (Fig 5F). As shown in other parts of this work, the persistence component measured by the new procedure is also not affected by the wound stimulus, even in presence of an increased bias component, and remains within levels close to about half the random module, both in presence (Fig 5G) and in absence (Fig 5C) of wound; it is also not changed when, during the observation time, the wound starts to close.

**Fig 6:**
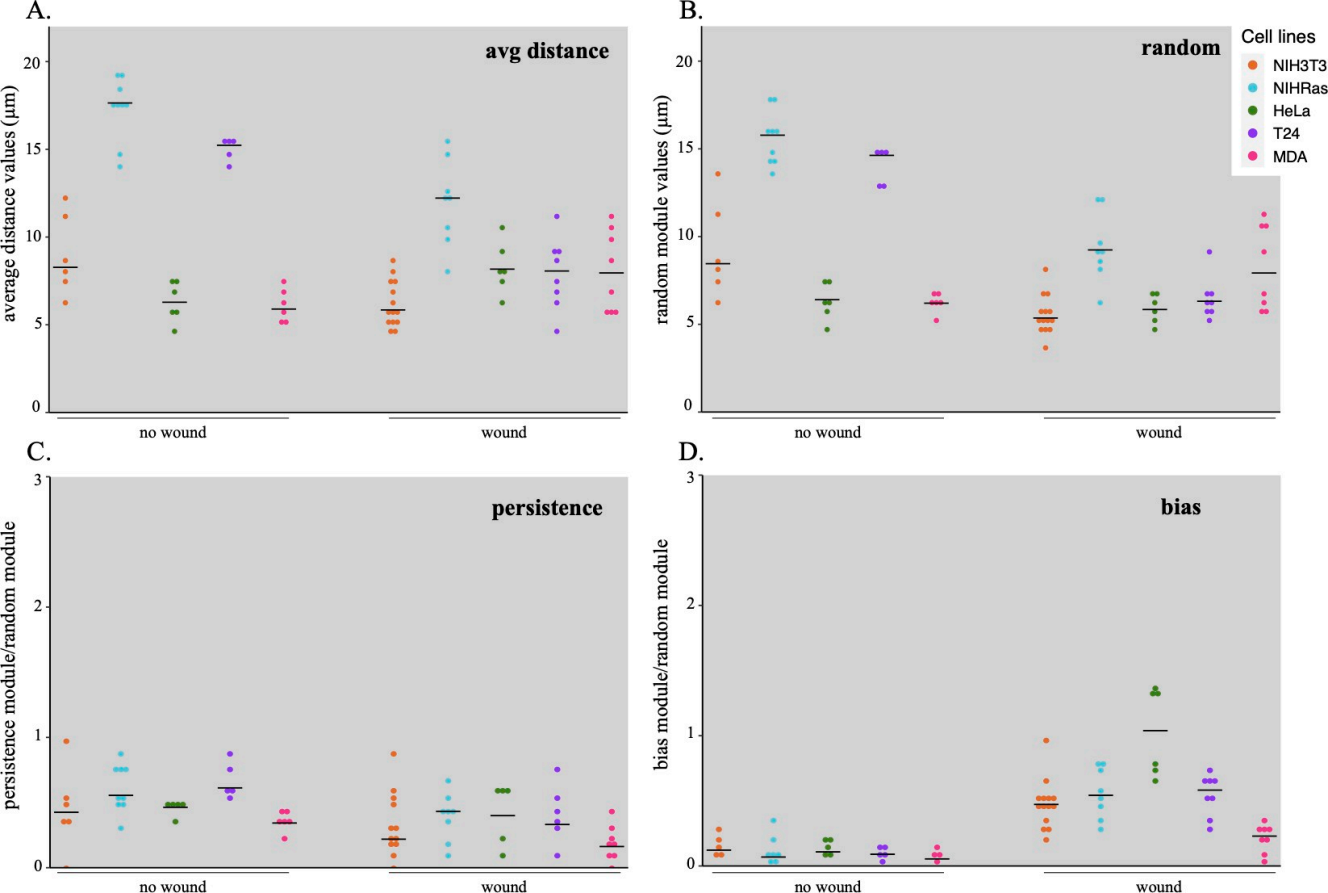
Movement of different cell lines in wound healing experiments. NIH-3T3 (orange), NIH-Ras (blue), HeLa (green), T24 (violet) and MDA-MB-231 (magenta) cell lines were grown on a culture plate and their movement was followed in both standard condition (no wound) and after stimulation (wound) by a wound inflicted to the cell layer. (A) Average distance and (B) random module, (C) persistence and (D) bias. Values reported in (C) and (D) have been normalized against the corresponding random modules. For each cell line, coloured points correspond to independent cultures analysed over a 4 hour time window, while their median value is reported as a small horizontal black trait.

In Fig 6, the presented method was used to comparatively study the motion properties of HeLa cells as well as four additional cell lines, NIH-3T3, NIH-Ras, T24 and MDA-MB-231. Average distance (Fig 6A) and random component (Fig 6B) are separately reported for each cell population, as well as persistence (Fig 6C) and bias (Fig 6D). Random components vary between different cell lines, being higher for NIH-Ras and T24 cell populations which also show higher average cell displacement modules. All cell lines respond to the wound stimulus with movement characterized by a strong bias component, clearly higher than that observed in its absence; in contrast, persistence is always present with values ranging between 0.4 and 0.8 times the random module and is not significantly modified in presence of a wound stimulus.

## Discussion

Eukaryotic cells in culture move according to migratory patterns that can often be described as a superdiffusive movement, typically associated with a degree of persistence and/or directional bias. These contributions to movement may be individually detected [5, 10, 28], but, at the best of our knowledge, the relative contribution of persistence and directionality to movement cannot be independently assessed by commonly used methods. The persistence model of Formula 2, for example, which is one of the most used, is particularly effective in interpreting superdiffusive motion patterns and allows for different degrees of persistence, with the preceding step influencing in different measure the direction taken in the next one. However, this straightening is always assumed to depend on the previous movement, rather than other external influences and therefore it is difficult to separate from similar effects, such as those induced by a global uniform directional bias. This happens, for example, when analysing cells moving under a wound stimulus and leads to inflated persistence values, as easily confirmed by analysis of migration patterns obtained from simulated cell populations (see Table 1, where datasets simulated with zero persistence show a measurable amount of persistence, and much larger values are seen if persistence is higher than zero). Similarly, independent bias evaluation makes the bias components appears larger than that used to generate movement, if persistence is introduced at the same time.

The method described here derives from attempts to analyse cell movement in terms of random, persistence and bias components, summed up into a vector that corresponds to the observed cell displacement; in this way, the ability of a cell to move (*random*), maintain motion direction (*persistence*) and respond to a directional stimulus (*bias*), may be independently assessed. When used to analyse cell populations, both wounded and unwounded, as well as *in silico* generated datasets, the method clearly distinguishes bias and persistence components within overall movement. Persistence evaluated in this way behaves as a feature of a given cell line and is not affected by directional bias as it remains relatively stable even when directional movement is stimulated in presence of a wound.

Although clearly improved if compared with simpler models, this approach has of course features which could limit its use in specific cases. When bias and persistence modules, are evaluated in this way, they include a scale component which depends on the length of cell displacement and may result in a more difficult comparison between cells with different motion features. This may be eased by making them independent of displacement length, by normalizing bias and persistence modules against the corresponding random module, as in Figs 4–6. An additional feature is that bias component, at least in principle, is assumed to be constant for all cells in different parts of the culture surface and during the analysis time. Adding support for other bias models would certainly be possible, although it would of course complicate the procedure.

The model proposed in this work assumes the random module to be the same for all steps and cells, while it could of course have different values for each path or cell. Analysis of the random modules of the tested populations showed a right long tail distribution, with median values close to, but lower than, the calculated mean random module of the population, as expected for this type of distribution. However, when aggregated by cell path, random modules showed a distribution similar to that of unaggregated ones, with average and median random modules typically differing by only a few units percent from those estimated on the unaggregated modules, as seen in S3 Fig.

Comparative analysis of motion paths followed by five different cell lines (Fig 6) reveals in all cases random and persistence vectors, with no or very low bias in unwounded cultures, that increases when a directional stimulus is introduced. Random and persistence components vary in the different cell lines, being higher for NIH-Ras and T24 cell populations, which show larger cell displacements. The contribution of each component to overall movement may vary in time, possibly reflecting the culture conditions, and changes in any component end up by affecting overall displacement. This may be useful to highlight movement trends within a given cell population: for example, in HeLa cells observed in wound healing experiments (Fig 5E–5H), average displacement tends to reduce in time. Since random and persistence components remain basically constant, this shortening is due to a progressive reduction of the bias module, which in turn is possibly related to the attenuated directional stimulus, determined by the disappearing empty space.

The method well supported the study of cell migration in experimental setups suitable to mimic wound repair in vitro and is promising to also be effective in other situations where migration is primed by stimuli such as local nutrient availability or presence of chemoattractants/chemorepellents. In principle, the model could be applied in all situations in which moving objects are detectable by imaging and tracked to obtain paths; embryogenesis, neuronal crest cell migration, chemotaxis, immune cell trafficking, tissue and wound repair, epithelial-mesenchymal transition, tumour invasion and metastasis are some of the areas where using the presented method could be helpful to better understand and predict migrating cell behaviour.

Finally, as for other mathematical models [28], the presented one may turn out to be useful in other fields of study, such as following animal migration and their response to environmental changes.

## Supporting information

S1 Table Random modules of simulated cell populations of Table 3. Random modules are measured for each simulated cell population reported in Table 3 of the Results, where the random input value is set to 9 μm/40min.
(TIF)

S2 Table Bias and persistence of synthetic cell populations. For each synthetic cell population, whose movement was simulated starting from a random module of 12.5 μm/40 min, the measured values of bias (*b*) and persistence (*p*) are compared with those expected (reported in header row and header column, respectively), i.e. those used as input data to generate the synthetic cell movement.
(TIF)

S3 Table Random modules of simulated cell populations of S2 Table. Random modules are measured for each simulated cell population reported in S2 Table, where the random input value is set to 12.5 μm/40min.
(TIF)

S1 Fig Time course of wound closure of HeLa cells. Phase contrast images represent the progression of empty space occupancy by HeLa cells at the time 0 and 6, 12 and 24 hours after wound.
(TIF)

S2 Fig Population size effect on the analysis with the proposed model. For simulated datasets of size ranging between 10 and 100 cells the estimated parameters are reported as ratio between calculated and expected values, the latter are indicated in each graph with distinct colours.
(TIF)

S3 Fig Density analysis of random module of analysed cell populations. Density analysis is applied on random modules from populations randomly moving (A-E) and under wound stimulus (F-J). (K-T) The same evaluation is performed on mean cell random modules.
(TIF)

S1 File Datasets used for cell population analyses and model testing. Text files containing information about displacements of experimental and simulated populations used for both preliminary analyses, in “datasets used for preliminary evaluation of cell movement (Tables 1 and 2)”, and further characterization of cell movement, in “further datasets used for cell movement characterization (Figs 2, 4–6; Table 2; S1–S3 Tables; S2 and S3 Figs)”.
(ZIP)

S2 File Pseudocode describing simulation steps. The file describes the steps followed at each time for each cell to simulated displacement.
(TXT)
