# Supplementary figures and images for: A three component model for superdiffusive motion effectively describes migration of eukaryotic cells moving freely or under a directional stimulus
Source: PLoS One. 2022 Aug 2;17(8):e0272259. doi: 10.1371/journal.pone.0272259 (PMC9345344; doi:10.1371/journal.pone.0272259)

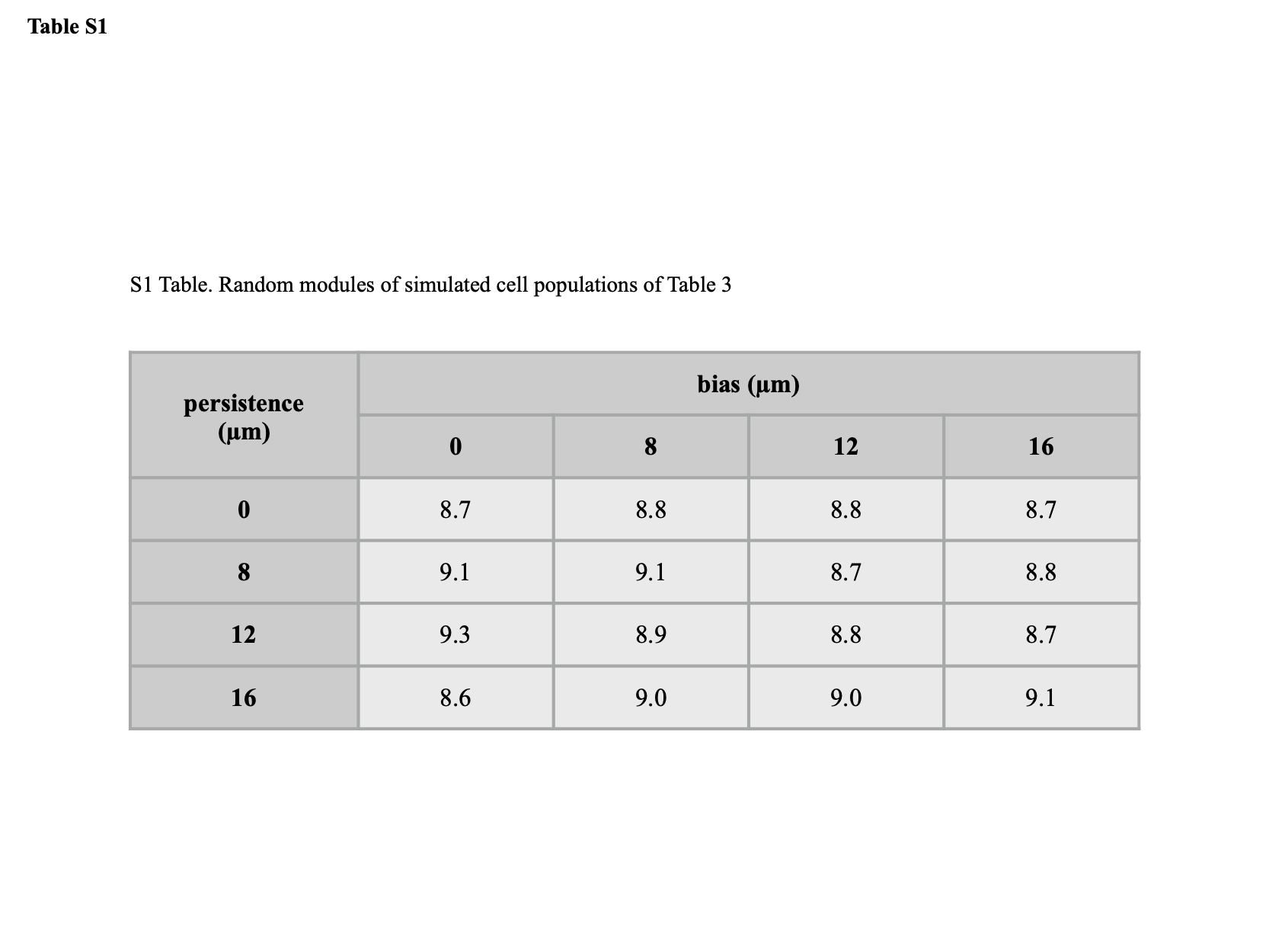

Supplement: S1 Table — Random modules are measured for each simulated cell population reported in Table 3 of the Results, where the random input value is set to 9 μm/40min. (TIF) [file pone.0272259.s001.tif]

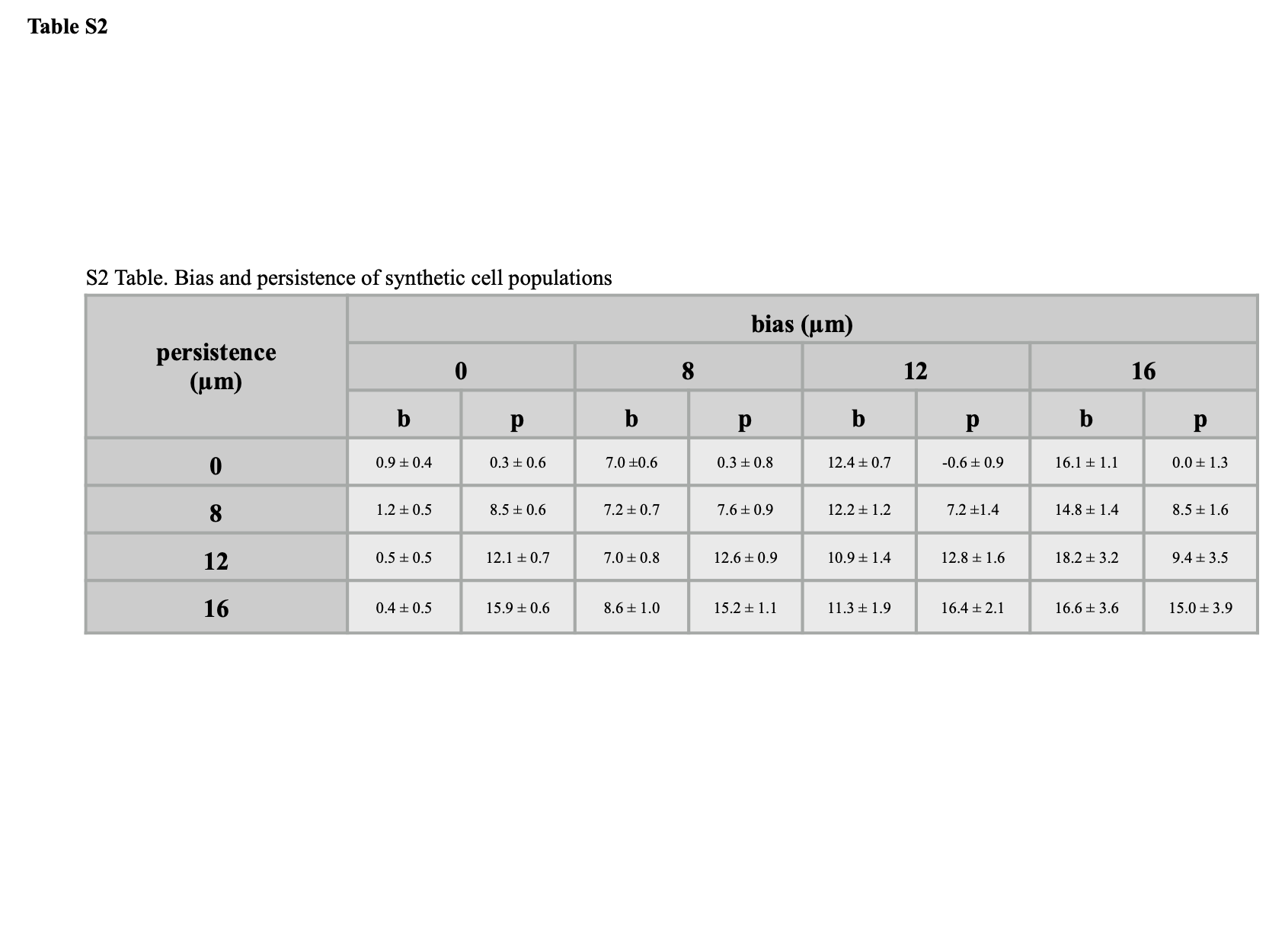

Supplement: S2 Table — For each synthetic cell population, whose movement was simulated starting from a random module of 12.5 μm/40 min, the measured values of bias (b) and persistence (p) are compared with those expected (reported in header row and header column, respectively), i.e. those used as input data to generate the synthetic cell movement. (TIF) [file pone.0272259.s002.tif]

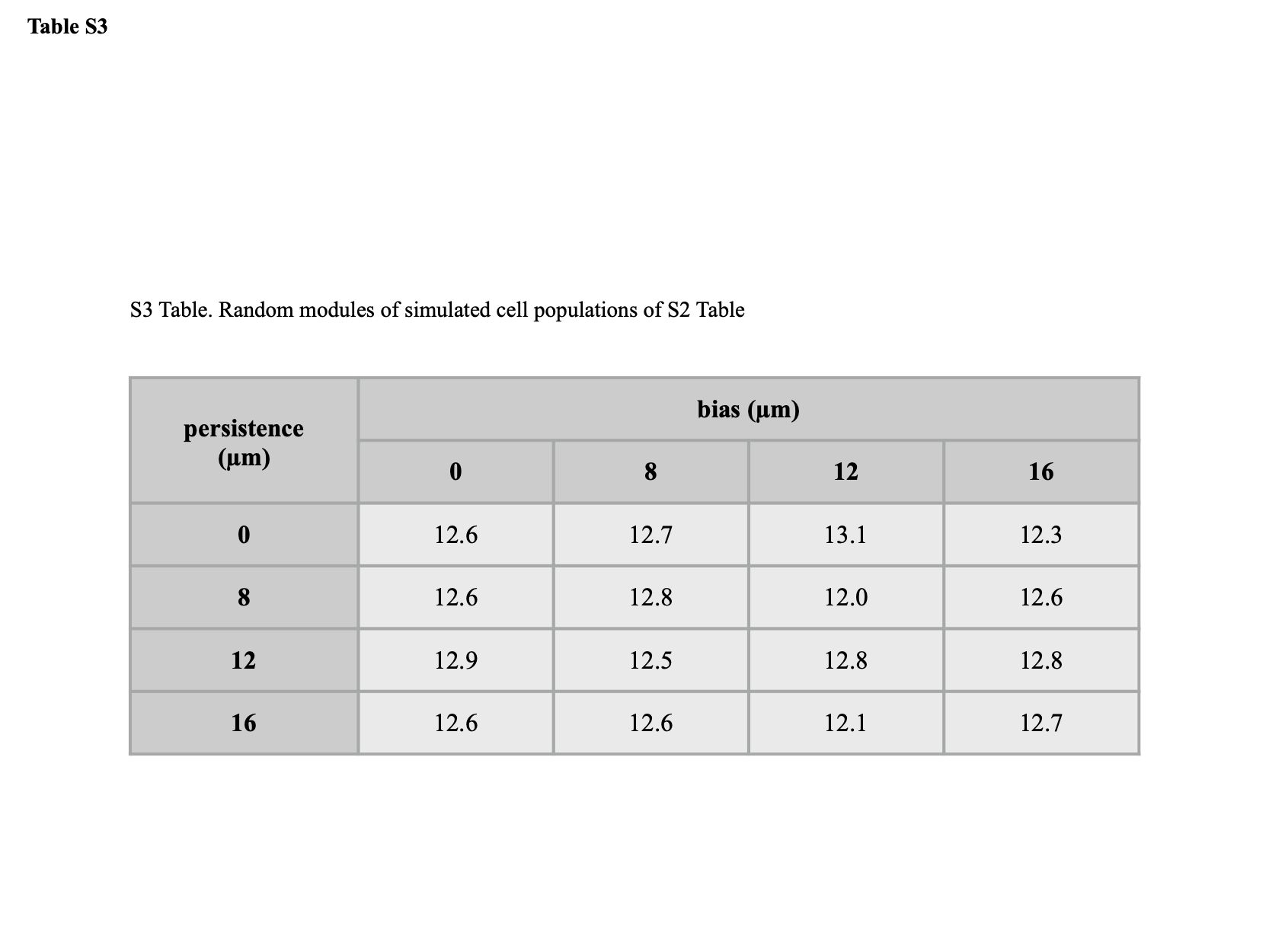

Supplement: S3 Table — Random modules are measured for each simulated cell population reported in S2 Table, where the random input value is set to 12.5 μm/40min. (TIF) [file pone.0272259.s003.tif]

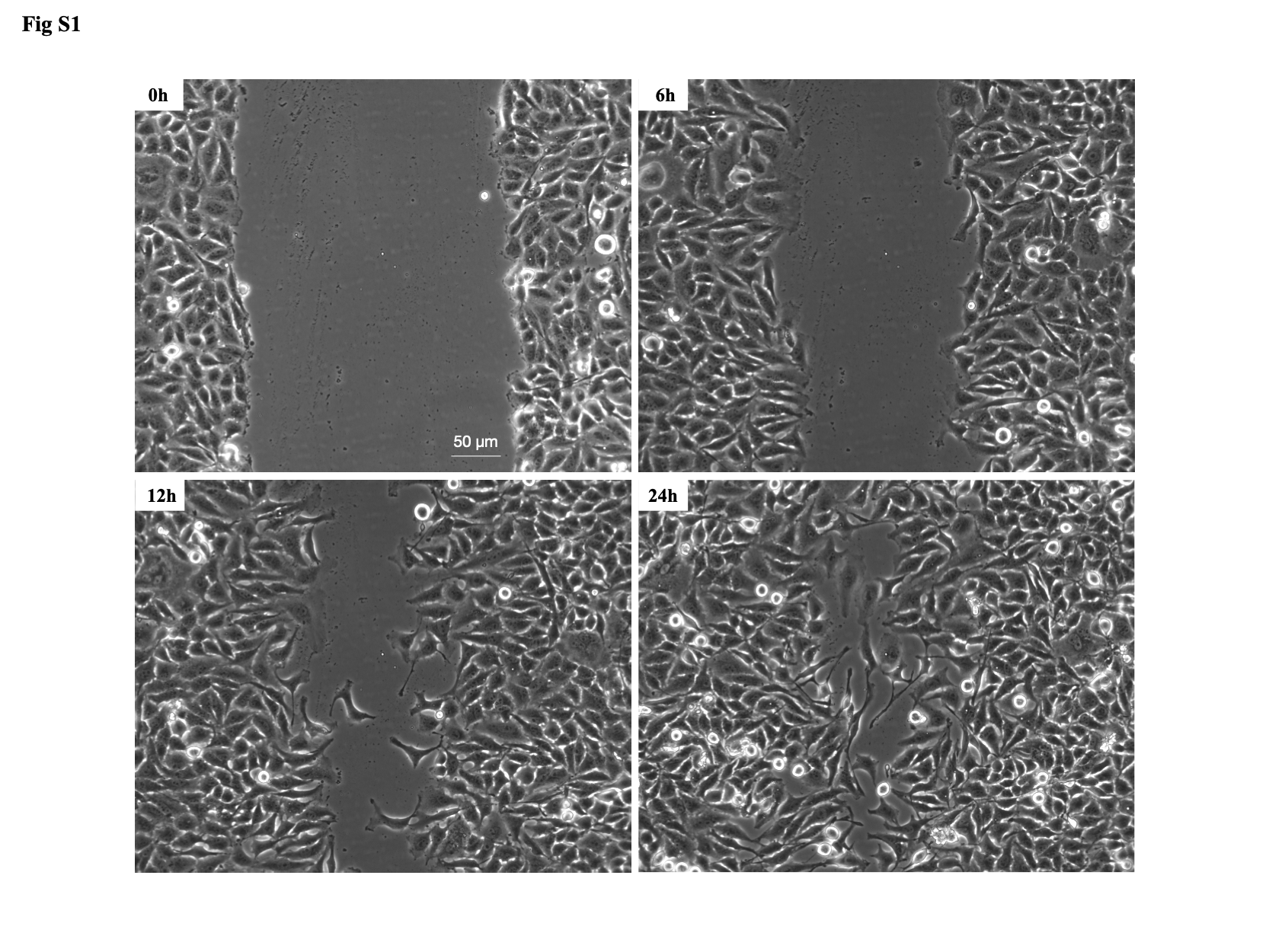

Supplement: S1 Fig — Phase contrast images represent the progression of empty space occupancy by HeLa cells at the time 0 and 6, 12 and 24 hours after wound. (TIF) [file pone.0272259.s004.tif]

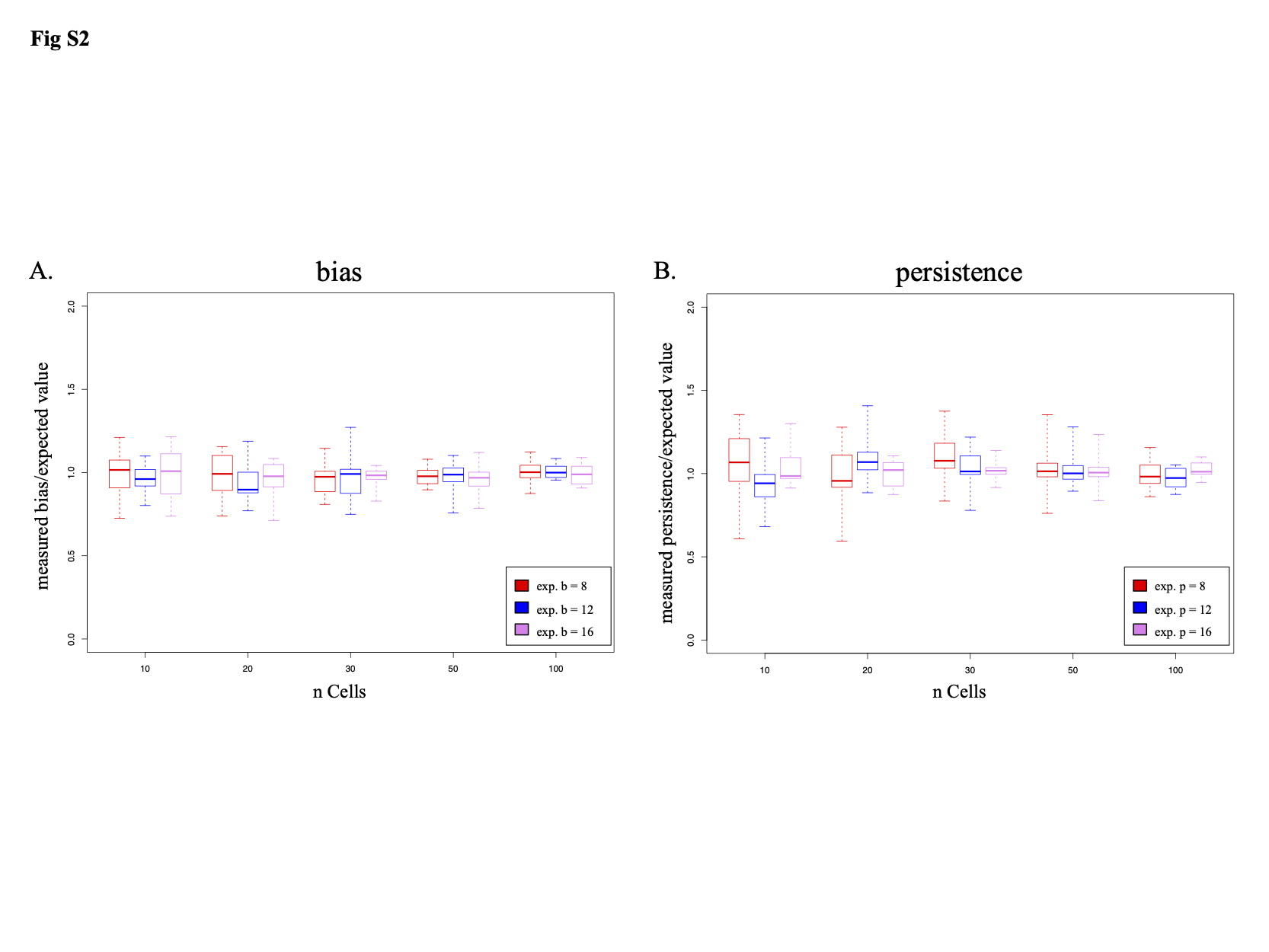

Supplement: S2 Fig — For simulated datasets of size ranging between 10 and 100 cells the estimated parameters are reported as ratio between calculated and expected values, the latter are indicated in each graph with distinct colours. (TIF) [file pone.0272259.s005.tif]

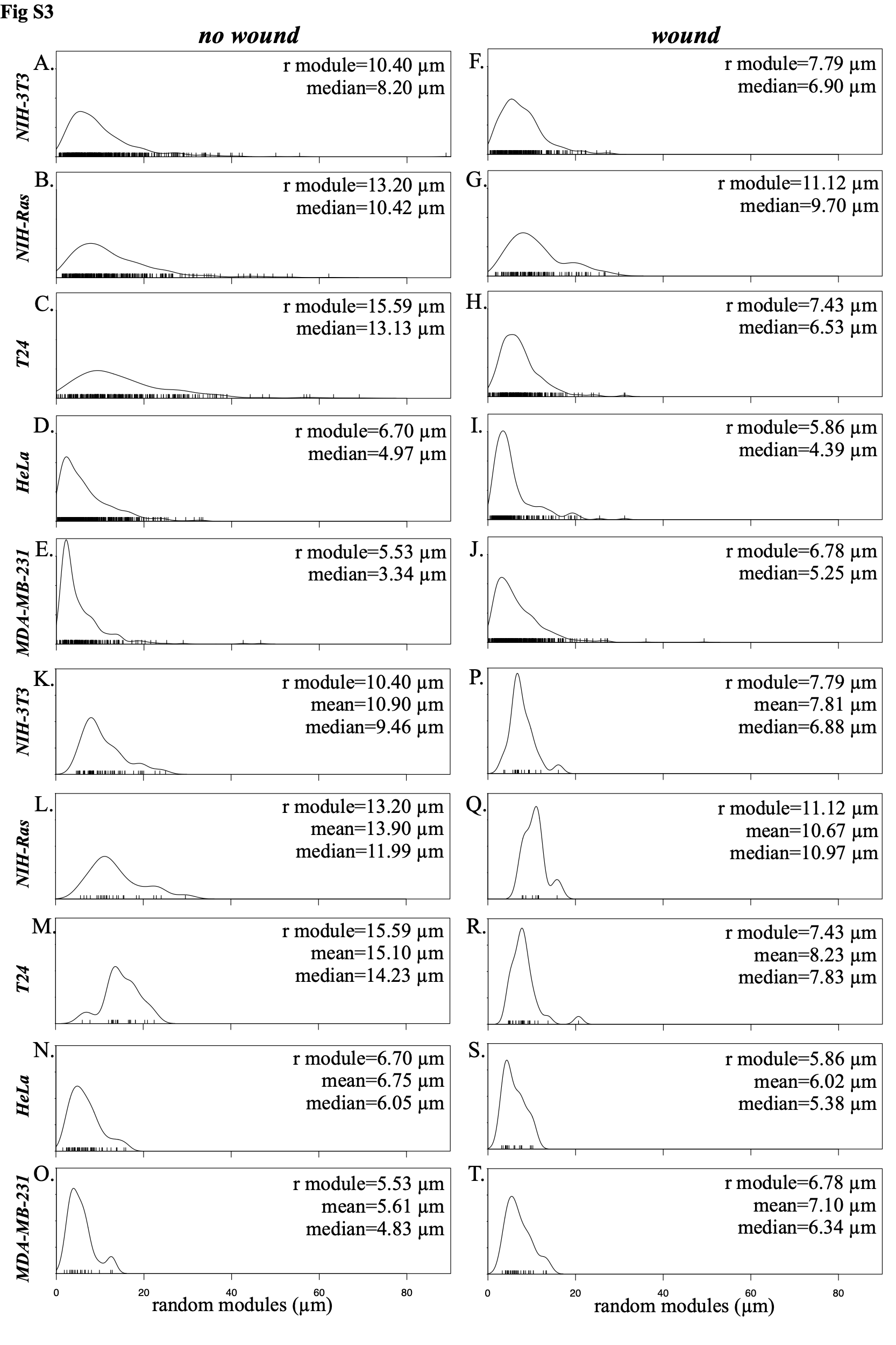

Supplement: S3 Fig — Density analysis is applied on random modules from populations randomly moving (A-E) and under wound stimulus (F-J). (K-T) The same evaluation is performed on mean cell random modules. (TIF) [file pone.0272259.s006.tif]
